# High Titers of*Chlamydia trachomatis* Antibodies in Brazilian Women with Tubal Occlusion or Previous Ectopic Pregnancy

**DOI:** 10.1155/2007/24816

**Published:** 2007-05-17

**Authors:** A. C. S. Machado, E. M. B. Guimarães, E. Sakurai, F. C. R. Fioravante, W. N. Amaral, M. F. C. Alves

**Affiliations:** ^1^Department of Microbiology, Immunology, Parasitology, and Pathology, Institute of Tropical Pathology and Public Health, Federal University of Goiás, Rua Delenda Resende de Mello S/N, 74605-050 Goiânia, Goiás, Brazil; ^2^Division of Adolescent Medicine, Department of Pediatrics, Faculty of Medicine, Federal University of Goiás, Setor Universitário, 74233-180 Goiânia, Goiás, Brazil; ^3^Department of Statistics, Federal University of Minas Gerais, Rua Juiz de Fora 801, 30180-060 Belo Horizonte, Minas Gerais, Brazil; ^4^Department of Gynecology and Obstetrics, Faculty of Medicine, Federal University of Goiás, Setor Universitário, 74233-180 Goiânia, Goiás, Brazil

## Abstract

*Objective*. To evaluate serum chlamydia antibody titers (CATs) in tubal occlusion or previous ectopic pregnancy and the associated risk factors.*Methods*. The study population consisted of 55 women wih tubal damage and 55 parous women. CAT was measured using the whole-cell inclusion immunofluorescence test and cervical chlamydial DNA detected by PCR. Odds ratios were calculated to assess variables associated with*C. trachomatis* infection.*Results*. The prevalence of chlamydial antibodies and antibody titers in women with tubal occlusion or previous ectopic pregnancy was significantly higher (*P* < .01) than in parous women. Stepwise logistic regression analysis showed that chlamydia IgG antibodies were associated with tubal damage and with a larger number of lifetime sexual partners.*Conclusions*. Chlamydia antibody titers were associated with tubal occlusion, prior ectopic pregnancy, and with sexual behavior, suggesting that a chlamydia infection was the major contributor to the tubal damage in these women.

## 1. INTRODUCTION

*Chlamydia trachomatis* is the most common bacterial sexually transmitted infection worldwide, especially among young adults [1]. Chlamydia infections remain often undiagnosed, as they are asymptomatic in the majority of patients. Undiagnosed and untreated chlamydia infections can ascend to the upper genital tract, where they colonize the endometrial mucosa and the fallopian tubes, leading to pelvic inflammatory disease (PID). Chlamydial PID can cause tubal occlusion and subsequent infertility, or partial occlusion with an increased risk for ectopic pregnancy [2]. However, most women who have tubal infertility or ectopic pregnancy have never been diagnosed with*C. trachomatis* PID because their infections have been asymptomatic or subclinical.

Risk factors frequently associated with chlamydial PID and its sequelae are young age, sexual intercourse at an early age, a large number of sexual partners, inconsistent condom use, and the presence of chlamydia antibodies [3].

Seroepidemiological studies have indicated that chlamydia infections account for a large proportion of asymptomatic genital tract infections by demonstrating a strong link between tubal pathology and the presence of chlamydia antibodies [4,5]. Thus, chlamydia IgG antibodies are associated with the development of late sequelae and are markers for previous exposure or endogenous reactivation of a previous chlamydia infection. In chronically infected patients negative for endocervical*C. trachomatis*, a positive serological test may be the only indication of chlamydia involvement [6].

In the present study, we evaluated whether prior exposure to*C. trachomatis*, as determined by IgG chlamydia antibody titers, was associated with infertility due to tubal occlusion and ectopic pregnancy in Brazilian women.

## 2. SUBJECTS AND METHODS

### 2.1. Study population

The study was performed in subfertile and parous women who entered a clinic specialized in human reproduction in the municipality of Goiânia, a city with 1 093 007 inhabitants in the central region of Brazil. During the period from March to December 2001, 110 women aged 18 to 38 were selected. Group I consisted of 33 women with infertility due to unilateral or bilateral tubal occlusion, confirmed by laparoscopy, and 22 women that presented one or more prior episodes of ectopic pregnancy. The group II consisted of 55 parous women.

### 2.2. Data collection and blood samples

Women who had used oral or topical vaginal antimicrobial treatments 15days prior to sample collection were excluded from the study. After giving written consent, women underwent standardized interviews concerning demographic characteristics, gynecological and obstetrics antecedents, self behaviors, potential risk factors for cervical and vaginal infections and symptoms. Peripheral blood was collected and all sera were transported, under refrigerated conditions, to the Laboratory of Cellular Immunology, Institute of Tropical Pathology and Public Health of the Federal University of Goiás, where they were cryopreserved at −20°C.

### 2.3. Endocervical sample collection

The gynecological examination included a careful examination of the abdomen, external and internal genitalia, and a speculum examination was performed. After cleaning the ectocervix, collection proceeded with an appropriate swab, which was introduced into the endocervix and then rotated for five seconds and removed carefully. It was immediately placed in a PCR transportation tube and agitated for five seconds. The samples were transported to the laboratory, where they were at 2–8°C until processing, which occurred within 7days.

### 2.4. PCR for C. trachomatis

*C. trachomatis* DNA was amplified using the Amplicor kit (Roche Molecular Systems, Branchburg, NJ, USA), according to the manufacturer's instructions. The internal control was used in each amplification reaction, such as positive and negative controls to*C. trachomatis,* purchased by the kit.

### 2.5. Serological methods

Serum samples were assayed for chlamydia IgG antibodies employing the Hemagen Virgo*C. trachomatis* IgG test (Electronucleonics Incorporation, Columbia, Ill, USA), according to the manufacturer's instructions. This is a whole cell inclusion immunofluorescence assay (WIF) that uses L2 serotype of*C. trachomatis*. Positive reactions, inclusions presenting a brilliant apple green fluorescence, were identified with the aid of a fluorescence microscope (Olympus Vanox with a B2 filter) at 400× magnification. For a quantitative determination, serial dilutions in PBS were performed. Dilutions of sera were expressed as antibody titers from 1/16 to 1/4096, or negative (<1/16).

## 3. DATA ANALYSIS

Data processing and analyses were realized using the software programs Epi-Info 6.0 (CDC, Atlanta, Ga, USA) and SPSS 8.0 (SPSS, Chicago, Ill, USA). Initially, a descriptive analysis of the main sociodemographic characteristics, sexual behavior of the participants, and its related risk factors was performed. The prevalence of*C. trachomatis* antibodies was calculated with corresponding 95% confidence interval (CI) and compared between groups by the*χ* ^2^ test or the Fischer exact test. For comparison of chlamydia IgG antibody titers, the Mann-Whitney test was used. Univariate and multivariate logistic regression analyses were performed to determine the risk factors associated with chlamydial antibodies.*P* < .05 were considered statistically significant.

## 4. APPROVAL

The study protocol was approved by the Ethics Committee on Human and Animal Medical Research of the University Hospital, Federal University of Goiás (Protocol no. 047/2001).

## 5. RESULTS

The demographic characteristics and sexual history of the study population are outlined inTable 1. The mean age ± the standard deviation for group I was 30.7 ± 4.3 years, while for group II it was 34.0 ± 4.6 years. Among the 55 women from group I, the great majority were married/cohabiting (87.3%); the same was observed in group II (81.8%). More than 2/3 of the population of groups I (91.0%) and II (76.3%) were educated to high school or university level, either complete or incomplete, which indicates a good level of education in this population. In relation to sexual behavior, the mean age for initiating sexual intercourse in group I was 19.4 ± 3.7 years old and for group II, 18.4 ± 2.7 years old. Seventeen women from group I (30.9%) and 11 from group II (20.0%) reported having four or more partners in life.

The prevalence of chlamydia IgG antibodies was significantly higher (*P* < .01) in the group I (31/55–56.4%) when compared to group II (17/55–31.0%). In the women with tubal occlusion this value was 54.5% and in those with previous ectopic pregnancy it was 59.1% (*P* > .05). Nine women in the group I (16.4%) reported a previous PID episode. The clinical diagnosis for PID was based on the following criteria: acute pelvic pain, especially in the postmenstrual period, fever, abnormal cervical discharge, uterine/adnexial tenderness, and cervical motion tenderness. Eight of these women (88.9%) presented with chlamydia IgG antibodies.

**Figure 1:**
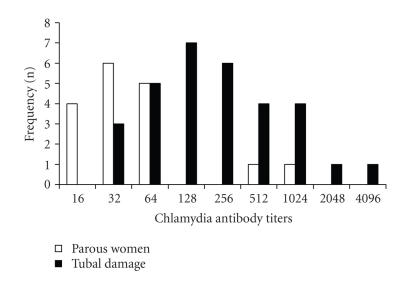
Frequency of*C. trachomatis* antibody titers in women with tubal damage and in parous women.

Among the 31 positive samples from group I, 23 (74.2%) presented titers greater than 1/64, while in group II this occurred in only two (11.8%) of the 17 positive samples (*P* < .01) (Figure 1). Moreover, titers equal to or greater than 1/128 were found in 42.4% of the samples of women with tubal occlusion and in 40.9% of those with previous ectopic pregnancy (*P* < .05). Titers ≥1/1024 were found in six patients (21.4%) and two of them were positive to*C. trachomatis* plasmid DNA.

*C. trachomatis* DNA was only detected in two endocervical samples from group I (3.6%; IC 95%) and in none of the 55 samples from group II.

Univariate analysis was performed to determine the degree of association between sociodemographic findings and sexual behavior and the presence of chlamydia antibodies (Table 2). The odds ratios (OR), with their respective 95% confidence intervals (CI) showed statistical significance in group I for the variables: number of sexual partners (2 to 3 partners compared to 1; ≥4 compared to 1) and previous PID. In group II, only the previous STD variable showed significant association.

After adjustment to a logistic regression model, chlamydia IgG antibodies were significantly associated with a greater number of lifetime sexual partners (estimated OR 3.06; CI 95% 1.7–5.5;*P* < .05) and with tubal pathology (estimated OR 2.93; CI 95% 1.2–6.9;*P* < .05).

## 6. DISCUSSION

In the current study, we found a high prevalence rate and titers of chlamydia IgG antibody among women with tubal occlusion or previous ectopic pregnancy from the central part of Brazil and an association between chlamydial antibodies and a higher number of lifetime sexual partners.

The prevalence rate of chlamydia IgG antibodies was significantly higher (56.4%) in the subfertility group than in parous women (31.0%). These results are in agreement with other studies that used immunofluorescence, like that of Kihlström et al. [7], who found chlamydia IgG antibodies in 56% of women with previous PID, tubal factor infertility, or prior ectopic pregnancy. Other authors have also reported high levels of chlamydia antibodies in women with tubal pathology, using other diagnostic tests [8].

Chlamydia antibody titers (CATs) have been shown to be of predictive value in the detection of tubal damage and increased risk of ectopic pregnancy [4,9,10] and are quantitatively related to the severity of damage [6]. These observations are in agreement with our results. Titers equal to or greater than 1/128 were found in around 40% of the women with tubal occlusion or previous ectopic pregnancy and in six patients the titers were ≥1/1024 (21.4%).

The immunofluorescence test employed in the present study is highly sensitive, as shown by a blinded comparative study of other serological tests for*C. trachomatis* antibody carried out in two international centres [11,12]. The test detects both group-specific lipopolysaccharide and species-specific antibodies [13]. Therefore, women with a positive serology but with a normal pelvis may have cross-reactive responses to past infection with other species of chlamydia such as*Chlamydia pneumoniae* [14] or*Chlamydia psittaci* [12,15]. In our study we did not evaluate*C. pneumoniae* antibodies. However, den Hartog et al. [6] evaluated serum of women with distal tubal pathology (DTP) and found that the presence of*C. trachomatis* antibodies was the only independent predictor for DTP. The predictive value of*C. trachomatis* antibodies for DTP could not be improved by adding test results of *C. pneumoniae* or lipopolysaccharide antibody testing.

Based on the correlation between chlamydia IgG antibody titers and the presence of tubal sequelae, some authors suggest that testing for these antibodies should be part of the basic routine investigation in infertility clinics [5,16]. Land et al. [10] have incorporated this procedure into the routine of their services since 1992. The predictive value of chlamydia IgG antibody titers detected by indirect immunofluorescence in the diagnosis of tubal pathology is considered by some to be comparable, or even superior to, that of histerosalpingography, according to some authors [17,18].

An association between the presence of chlamydia IgG antibodies and the number of sexual partners occurred in this study. This finding is in agreement with previous studies which show that this is one of the most relevant risk factors in the acquisition of PID and its sequelae [19,20].

In the current study, chlamydia IgG antibodies were also an important risk factor for tubal occlusion and ectopic pregnancy. After adjustment to a multivariate model, a frequency of 2.9-fold greater exposure to*C. trachomatis* in subfertile women was found in comparison with parous women. These data suggest a positive association between chlamydia infection and the risk of developing ectopic pregnancy and tubal infertility. In a previous study, it was shown that exposure to this pathogen is approximately three-fold greater in women with tubal infertility and ectopic pregnancy in comparison with a control group of fertile women [21].

Only two women in the present study were positive for *C. trachomatis* in their endocervix by PCR. Other authors have also found low prevalence rates of 1.3–8.3% in similar populations, using nucleic acid amplification techniques [11,22,23]. One possible explanation for this low chlamydia detection rate could be that in women with tubal infertility or with a previous ectopic pregnancy the bacterium has ascended through the cervix and endometrium to the fallopian tubes and is no longer present in the endocervix. This hypothesis is supported by the data of Patton et al. [24]. The authors found*C. trachomatis* DNA and/or antigens in fallopian tubes from 19 of 24 women (79.2%) with tubal factor infertility suggesting a persistent upper genital tract chlamydial infection. Furthermore, evidence exists that*C. trachomatis* may persist in a viable and metabolically active state in the upper genital tract, despite negative PCR results from the cervix. Possible reactivation of the microorganism, for example by uterine instrumentation, may result in a renewed upper genital tract infection [25].

In conclusion, we demonstrated a high prevalence and titers of chlamydia IgG antibody in Brazilian women with tubal occlusion or prior ectopic pregnancy. Chlamydia antibodies were associated with sexual behavior. Thus, an inapparent chlamydia infection could have triggered these sequelae in the studied population confirming the importance of*C. trachomatis* as a cause of tubal dysfunction in this population. For practical clinical purposes, chlamydia serology is useful mainly as a screening test for the likelihood of tubal damage in infertile women and may facilitate decisions on which women should proceed with further more invasive investigations. Our data reinforce the need for public health policies in developing countries, which promise triage and eventual treatment for young women with*C. trachomatis* genital infections, thus avoiding the serious sequelae to women's reproductive health and a reduction in the financial burden hospital commitment.

## Figures and Tables

**Table 1:** Sociodemographic characteristics and sexual behavior of 55 women with tubal occlusion or previous ectopic pregnancy (group I) and 55 parous women (group II), from Goiânia, Goiás, Brazil, in 2001.

Variable	Group I n (%)	Group II n (%)

**Marital status**		
Married/cohabiting	48 (87.3%)	45 (81.8%)
Single	07 (12.7%)	10 (18.2%)

**Schooling, y**		
<5	—	02 (3.6%)
5–8	05 (9.1%)	11 (20.0%)
9–11	25 (45.5%)	29 (52.7%)
>11	25 (45.5%)	13 (23.6%)

**1st sexual intercourse, y**		
≤15	07 (12.7%)	11 (20.0%)
>15	48 (87.3%)	44 (80.0%)

**Number of partners**		
1	20 (36.4%)	22 (40.0%)
2–3	18 (32.7%)	22 (40.0%)
≥4	17 (30.9%)	11 (20.0%)

**Previous STD**		
Yes	04 (7.3%)	04 (7.3%)
No	51 (92.7%)	51 (92.7%)

**Previous STD partner** ^*^		
Yes	04 (7.3%)	02 (4.4%)
No	44 (91.7%)	43 (95.6%)

**Previous PID**		
Yes	09 (16.4%)	—
No	46 (83.6%)	55 (100.0%)

**Relations with symptomatic partner** ^*^		
Yes	05 (10.4%)	03 (6.7%)
No	43 (89.6%)	42 (93.3%)

^*^These items were only replied to by women married or in consensual union.

**Table 2:** Univariate analysis of the characteristics associated with the presence of chlamydia IgG antibodies in women with tubal occlusion or previous ectopic pregnancy (group I, *n* = 55) and in parous women (group II, *n* = 55), in Goiânia, Goiás, Brazil, in 2001.

Variable	Group I IgG+	OR	IC 95%	Group II IgG+	OR	IC 95%

**Age, y**						
≤30	18 (58.1%)	2.31	(0.7–8.0)	05 (29.4%)	2.75	(0.5–13.9)
>30	13 (41.9%)			12 (70.6%)		

**Marital status**						
Married/cohabiting	06 (19.4%)			14 (82.4%)		
Single	25 (80.6%)	5.52	(0.6–131.2)	03 (17.6%)	1.05	(0.2–6.1)

**Schooling, y**						
≤8	04 (12.9%)	3.41	(0.3–86.0)	03 (17.6%)	0.60	(0.1–2.9)
>8	27 (87.1%)			14 (82.4%)		

**N^º^ of sexual partners in life**						
≥4	14 (45.2%)	18.67	(2.9–150.1)^*^	06 (35.3%)	4.08	(0.7–26.3)
2 or 3	13 (41.9%)	10.40	(1.9–65.2)^*^	06 (35.3%)	1.27	(0.3–6.2)
1	04 (12.9%)			05 (29.4%)		

**Age at first sexual intercourse, y**					
≤15	06 (19.4%)	5.52	(0.6–131.2)	04 (23.5%)	1.36	(0.3–6.6)
>15	25 (80.6%)			13 (76.5%)		

**Previous STD**						
Yes	03 (9.7%)	2.46	(0.2–65.9)	03 (17.6%)	7.93	(0.6–216.4)^*^
No	28 (90.3%)			14 (82.4%)		

**Previous STD partner**						
Yes	02 (8.0%)	0.91	(0.1–10.2)	01 (7.1%)	2.31	(0.0–93.2)
No	23 (92.0)			13 (92.6%)		

**Previous PID**						
Yes	08 (25.8%)	8.00	(0.9–184.5)^*^			
No	23 (74.2%)					

**Relation with symptomatic partner**					
Yes	03 (12.0%)	1.43	(0.2–13.9)	01 (7.1%)	1.12	(0.0–18.2)
No	22 (88.0%)			13 (92.9%)		

^*^ *P* < .05.
